# Supplementary material for: Comparative analysis of novel hormonal agents in non-metastatic castration-resistant prostate cancer: A Taiwanese perspective
Source: PLoS One. 2024 Aug 7;19(8):e0306900. doi: 10.1371/journal.pone.0306900 (PMC11305548; doi:10.1371/journal.pone.0306900)
Supplement: Attachment — Submitted filename: Response to Reviewers.docx [file pone.0306900.s002.docx]

Dear reviewer #1

We had revised our manuscript along with your suggestions as list below. We sincerely appreciate for your advices.

1. Unmet Need Highlight:

In the background section, the authors should address the unmet need more explicitly. Specifically, they could emphasize the lack of direct head-to-head comparisons among the three FDA-approved ARPis (darolutamide, apalutamide, and enzalutamide). Highlighting this gap would strengthen the rationale for their study.

Answer: Thanks for your valuable comments. We have rewritten the background section, including the abstract and manuscript, to emphasize the rationale for this study.

2. Clarification on State Description:
o In line 21-22, the sentence mentions: “Non-metastatic castration-resistant prostate cancer (nmCRPC) refers to this state, which presents a potential threat to life.” However, the specific state is not clearly described. Please rephrase this sentence to provide clarity.

Answer: Thanks for your valuable comments. We have rewritten this section to address the definition of nmCRPC, the importance of PSADT, and the treatment goals for nmCRPC. (Page 5, line 17-25)

3. Support for Chemotherapy Use:
o Line 35-36 states: “Furthermore, when compared to chemotherapy, these treatments have demonstrated effectiveness in averting severe adverse effects [2, 7].” It’s essential to verify whether the cited references or guidelines indeed support the use of chemotherapy in nmCRPC status. I recommend asking the authors to reiterate this statement with appropriate evidence.

Answer: Thanks for your valuable comments. We have removed the description about chemotherapy for nmCRPC.

4. Replace “Review” with “Study”:
o In line 67, consider replacing “review” with “study” to avoid any confusion. This change will prevent readers from mistaking the article as a review article.

Answer: Thanks for your valuable comments. We have replaced all instances of "review" with "study" to avoid any confusion and ensure clarity..

5. Clarification on nmCRPC Definition:
o Lines 104-107 discuss nmCRPC status. However, the usual definition excludes previously identified radiologically evident metastasis. The statements in this section seem to deviate from the standard definition. Please ask the authors to clarify this point for better alignment.

Answer: Thanks for your valuable comments. We further clarified this clinical situation with the description: “According to the original definition of nmCRPC, these patients should have been excluded. However, in clinical practice, when we apply for medication treatment, the lack of metastatic lesions means we can only follow the treatment options for nmCRPC”. (Page 10, line 93-98)

Dear reviewer #2

We had revised our manuscript along with your suggestions as list below. We sincerely appreciate for your advices

1. Think about demonstrating the PSA or other character changes across various treatments for greater clarity with figures.

Answer: Thanks for your valuable comments. We have added a figure illustrating the PSA response data for various NHAs at different time points. Additionally, we have further clarified the PSA response on Page 14, lines 136-147.

2. Strengthen your conclusion by validating it with additional datasets..

Answer: Thanks for your valuable comments. We have revised the manuscript to include detailed PSA response data, thereby strengthening our conclusion.

3. Please carefully review and correct any typos and grammar errors.

Answer: Thanks for your valuable comments. We have carefully reviewed the manuscript and corrected all typos and grammatical errors using ChatGPT.
